# Where and when to start: Regulating DNA replication origin activity in eukaryotic genomes

**DOI:** 10.1080/19491034.2023.2229642

**Published:** 2023-07-19

**Authors:** Clare S.K. Lee, Matthias Weiβ, Stephan Hamperl

**Affiliations:** Chromosome Dynamics and Genome Stability, Institute of Epigenetics and Stem Cells, Helmholtz Zentrum München, Munich, Germany

**Keywords:** 3D Genome Organization, chromatin structure, DNA replication, origin clustering, origins of replication, replication timing, histone modifications

## Abstract

In eukaryotic genomes, hundreds to thousands of potential start sites of DNA replication named origins are dispersed across each of the linear chromosomes. During S-phase, only a subset of origins is selected in a stochastic manner to assemble bidirectional replication forks and initiate DNA synthesis. Despite substantial progress in our understanding of this complex process, a comprehensive ‘identity code’ that defines origins based on specific nucleotide sequences, DNA structural features, the local chromatin environment, or 3D genome architecture is still missing. In this article, we review the genetic and epigenetic features of replication origins in yeast and metazoan chromosomes and highlight recent insights into how this flexibility in origin usage contributes to nuclear organization, cell growth, differentiation, and genome stability.

## Introduction

DNA replication is a fundamental process of every organism to duplicate the genome and provide an identical set of chromosomes to the two emerging daughter cells. This process is initiated at specialized DNA regions named origins of replication. Depending on genome size, eukaryotic cells utilize several hundred to thousands of replication origins to allow duplication of their large linear chromosomes in a manageable timescale. However, initiation across this large number of origins needs to be precisely coordinated in space and time to ensure accurate genome duplication during the confined S-phase of the cell cycle.

The initiation of DNA replication is partitioned into temporally discrete steps: During the G1 phase of the cell cycle, origins are first recognized by the hexameric origin recognition complex (ORC). Subsequently, the helicase-loader cell division cycle 6 (Cdc6) together with the chromatin licensing and DNA replication factor 1 (Cdt1) chaperone lead to the consecutive recruitment and head-to-head binding of two inactive minichromosome maintenance (MCM)2–7 hexamers onto the DNA. This completes origin licensing, the assembly of the inactive pre-replicative complex (pre-RC), and marks potential origins for firing in the following S phase. Upon entry into the S phase, cyclin-dependent kinases (CDKs) and Dbf4-dependent kinases (DDKs) are activated. These enzymes mediate, through multiple phosphorylation events of pre-RC components, the recruitment of cell division control protein 45 (Cdc45) and the Go-Ichi-Ni-San (5-1-2-3 in Japanese) (GINS) tetramer to the MCM2–7 hexamers to convert the inactive complex into the active CMG (Cdc45/Mcm2–7/GINS) helicase. After melting the DNA duplex by the MCM2–7 complex, DNA polymerases and additional replication factors assemble to activate the origin and generate a bidirectional replication fork. This separation of licensing and activation of origins is crucial to ensure once and only once genome replication per cell cycle (reviewed in [1] and [2]).

Another layer of complexity is added by the fact that only a subset of the licensed and thus initiation-competent origins will be activated by different cells in a stochastic manner [3–5]. Importantly, this excess of licensed origins provides a backup pool of dormant origins that can be activated under conditions of replication stress and assure timely genome duplication when replication forks are slowing or stalled [6]. This flexibility in origin usage also showcases the stochastic nature of replication initiation. Nevertheless, population-based studies that average these heterogeneous replication initiation events across a large number of cells can define a reproducible spatial and temporal program of chromosome replication [7,8]. This replication timing (RT) program is highly robust and evolutionary conserved from yeast to humans [9,10], suggesting that replicating different parts of the genome at different times has an important biological function. One likely reason is the need to coordinate DNA replication with other crucial processes such as transcription and DNA repair on the shared chromatin template to avoid DNA strand breaks and DNA damage [11–16]. This is also reflected by the fact that RT program changes can be observed during embryonic development [17], cellular differentiation [18], and various human diseases such as cancer [19].

The signal for early or late RT of individual origins is established in the G1 phase during origin licensing [20,21] and depends on the chromatin context of origins [22–24]. One critical feature of origin chromatin is the ability of licensed origins to compete for a stoichiometrically limiting pool of replication initiation factors [25–27]. In addition, a well-defined RT program ensures that the number of replication forks does not exceed the pool of available nucleotides and, therefore, consolidates productive elongation of replication forks, thereby preventing dormant origin firing, replication stress, and ensuring genome stability during cell division.

Individual origins also vary greatly in their efficiency which describes the probability of activation for each replication origin in a cell population [3]. Together, this plasticity of origin usage suggests that alternative, non-mutually exclusive mechanisms, such as chromatin features, nuclear organization, and cellular context, play roles in promoting or interfering with origin function. Here, we review our current knowledge about the genetic and epigenetic features of replication origins in eukaryotic genomes, with a particular focus on the differences and similarities of replication origins in yeast and metazoan genomes. We apologize to those colleagues whose work on archaeal and bacterial systems is not referenced due to space limitations but refer the reader to excellent reviews covering this topic [28–31].

## Replication origin sequence and initiator binding

**Figure 1.**
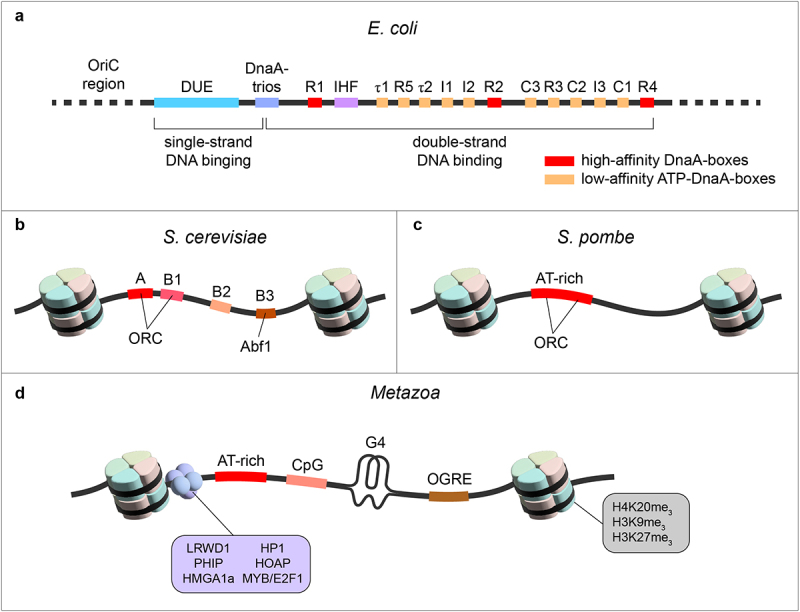
DNA sequence specificity of yeast and mammalian replication origin. (a) Illustration of sequence elements of *E. coli* replication origin (oriC). The DUE is flanked on one side by multiple high- and weak-affinity DnaA-boxes. Specific replication origin sequence elements for *S. cerevisiae* (b), *S. pombe* (c), and metazoan cells (d) are shown. DUE – DNA unwinding element, IHF – integration host factor, G4 – G quadruplex, OGRE – origin G-rich repeated element.

Almost six decades ago, Jacob, Brenner, and Cuzin proposed the replicon model to explain the regulation of chromosomal DNA synthesis in *E*. *coli* [32]. The model hypothesizes that a *trans*-acting factor, an initiator, interacts with a *cis*-acting DNA element, the replicator, to promote replication at a nearby origin. This concept laid the foundation for the quest to identify replicator DNA sequences and initiator proteins in prokaryotes and eukaryotes. Replicator DNA sequences were readily identified in prokaryotes, DNA viruses, and lower eukaryotes by their ability to maintain extrachromosomal plasmid replication, whereas initiators were identified based on their ability to bind their replicator sequence (Figure 1). These findings validated the model and quickly revealed the basic mechanisms of DNA replication by lower eukaryotic cells such as the budding yeast *Saccharomyces cerevisiae* [33].

### Autonomously replicating sequences (ARSs) serve as the yeast replicator

As mentioned, the search for replicators in budding yeast led to the identification of ARSs that support DNA replication of extrachromosomal DNA [34–36]. ARS regions are around 100–200 bp long and exhibit a multipartite nature containing A, B1, B2, and sometimes B3 elements that together play important roles in the origin function [37,38]. The A element contains the conserved 11-bp T-rich ARS consensus sequence (ACS) (WTTTAYRTTTW) [39,40], which, in conjunction with the B1 element, constitutes the primary binding site for the heterohexameric ORC, the eukaryotic replication initiator [38,41–43]. Interestingly, the ACS of individual origins is degenerate, and sometimes only a 10/11- or 9/11-bp match is needed for an initiation-competent ARS. In addition, most origins contain multiple imperfect matches to this motif with the best match not necessarily corresponding to the site of replication initiation. The functional importance of the ACS has also been shown in mutational analyses, where even single point mutations in highly conserved ACS positions could strongly decrease or even abolish the function of the replication origin [44]. But since there are over 12,000 ACS motifs in the yeast genome, of which only 300–400 are used under normal conditions, the presence of the ACS alone does not suffice to mark an active ARS, and it suggests that other chromatin features also take part in defining an origin. A less conserved, 17 bp long extended ACS (eACS) has later been identified, which has improved but still not sufficient power to predict the origin location in the yeast genome [45]. More recent bioinformatic approaches have now comprehensively identified most origins in several different yeast genomes with high accuracy [46–48], indicating to some degree the sequence-specific nature of yeast origins.

B1, B2, and B3 elements are collectively known as the B domain, which is located 3´ to the T-rich strand of the ACS and has been described as a DNA unwinding element, facilitating the access of replication factors to the DNA template [49–51]. The B1 element contains a conserved WTWDNA sequence motif 17 to 19 bp from the ACS [52] and acts together with the ACS as a bipartite DNA binding site for the ORC complex [38,43]. This was later referred to as ORC-ACS [53,54]. Recent structural studies of ORC bound to the origin DNA sequence showed that specific recognition of the ACS is carried out by a conserved basic amino acid motif of Orc1 in the minor groove and by a yeast-specific helical insertion motif of Orc4 in the major groove. Similar insertions into major and minor grooves in the B1 site induce ORC-specific bending of the DNA [42,55]. Interestingly, removing this insertion helix (IH) from Orc4 disrupts the ARS sequence-specific binding, thereby changing the selectivity of the ORC complex in yeast to randomly accessible regions in the genome with preference to transcriptional start sites [56].

The B2 DNA sequence has a similar consensus sequence ANWWAAAT as the ACS and has been suggested to function as a second ORC binding site under certain conditions or as a binding site for the Mcm2–7 double hexamers [57–61]. B3 contains a binding site for the transcription factor ARS binding factor 1 (Abf1) [62]. At certain origins, Abf1 binding might help to exclude nucleosomes from the ARS, thereby helping to establish a nucleosome-depleted region (NDR) [63]. However, B3 elements are not found within all budding yeast origins and Abf1 binding does not appear to be strictly essential for origin function [37,62,64].

In addition, a C domain located upstream of the A element was described. This domain can provide binding sites for transcription factors that can enhance origin activity, like MCM1 [65], SUppressor of Mar1-1 (Sum1) [66], and also origin binding factor 1 (OBF1) [67]. Again, mutations in these binding sites show an effect on origin function but are rather mild compared to mutations in the A or B domains.

Each of the ~300 annotated yeast origins shows distinct combinations in the distribution and number of ACS, eACS, B1, B2, and B3 elements, suggesting that they have auxiliary and partially redundant functions in origin specification. As most of these DNA elements interact with the core replication licensing machinery, a primary function of these motifs could be to efficiently guide ORC and MCM pre-RC complexes during origin licensing in G1-phase. This is also supported by a recent analysis showing that the presence or number of these individual DNA motifs shows no correlation with RT or efficiency of the origin [68], strengthening the hypothesis that the DNA sequence properties of individual origins allow efficient origin licensing in G1 phase but have no major impact on the RT and origin efficiency state in S phase.

### Metazoan replicators do not show specific sequence requirements

In metazoans, the search for specific chromosomal replicator sequences started with genetic assays and mutational analyses of potential replicator sequences to initiate replication at ectopic chromosomal locations [69–74]. Early studies in Xenopus egg extracts indicated that the loading of pre-RC complexes and replication initiation can occur randomly at any DNA sequence [75]. Later, despite substantial efforts using genome-wide mapping strategies of initiator binding or replication start sites to map origins in metazoans, little concordance is found among the studies and the identified replicator sequences do not display any clear consensus sequence [76–87] (see also Box 1 for more details). The most likely reason for this little concordance is the large difference in the resolution of individual methods used to map replication initiation sites, ranging from only a few kb to more than 30 kb. Furthermore, it is now widely accepted in metazoan cells that multiple replication origins cluster together in broader initiation zones (IZs), where multiple origins are present and fire randomly in a cell population. However, recent high-resolution short nascent strand (SNS)-Seq data are available and enhance the resolution of origin mapping in higher eukaryotic genomes [88–90].

Even in the few cases where mammalian origins have been mapped to relatively well-defined sequence elements, none of these crucial elements are able to specify initiation by themselves and they appear to fire with a very low efficiency of <10% [106]. For example, the Chinese hamster dihydrofolate reductase ori-β requires at least four elements for the initiation of DNA replication at ectopic loci [70] and the Drosophila chorion locus requires two distinct sequences: ori-β which is a segment that overlaps the replication origin and amplification control element 3, a replication enhancer that contains an essential 142-bp sequence [107,108]. Similarly, the human c-MYC replicator requires several elements [71] and the human β-globin locus contains two non-overlapping independent replicators in a close neighborhood within the transcribed locus [74]. Interestingly, this apparent modular organization of non-redundant DNA elements is potentially reminiscent of the control of yeast origins by different A, B, and C DNA motifs. However, the sequence motifs of such a metazoan collection of modules appear to be more diverse and much more dispersed up to several kilobases from the initiation site, resulting in the aforementioned broad IZs that span tens of kilobases and low firing efficiencies [109]. Therefore, the exact amount and location of origins in mammalian genomes are still an open question in the field [110].

However, certain sequence features are consistently detected across different origin mapping methods. While AT-richness defines ARS elements in yeasts, GC-rich sequences are more correlated with active replication initiation sites in metazoan genomes [78]. Interestingly, the high GC content can favor the formation of three-dimensional G-quadruplex (G4) structures that have been shown to impact origin efficiency [76,111–113]. These secondary structures have previously been linked to various genome features like nucleosome-free regions (NFRs) [114] or CpG islands [115]. Therefore, it is possible that these structures also play a role for replication origin selection and/or activity, but it remains controversial whether G4s positively or negatively influence DNA replication [116]. Interestingly, some mammalian origins are comprised of AT-rich DNA sequences that are upstream and downstream flanked by poly-dT and poly-dA tracts, respectively [117]. This AT-richness may represent another less frequent class of replication origins in mammalian cells that would strongly resemble the situation in yeast.

Although the specific locations of replication initiation sites across metazoan chromosomes are not fully resolved, the heterogenous nature of mammalian origins may be affected by the lack of sequence-specific binding of the mammalian ORC complex. Although *in vitro* studies suggest a weak affinity of human ORC for AT-rich sequences [118,119], no sequence preference is observed *in vivo* [85]. Interestingly, ORC shows an order of magnitude higher affinity for negatively supercoiled DNA than for relaxed DNA, which points to an additional mechanism for ORC selectivity toward the topological state of DNA [120]. G4 sequences in single-stranded DNA were also shown to preferentially bind ORC [121], and the presence of G4 motifs is positively correlated with high origin efficiency [77,113,122], suggesting that DNA secondary structures could play an important role in individual origins. In general, there is a clear preference for GC-rich regions in mammals [78,123]. Such CpG islands can also be found at active promoter regions, which could explain why replication origins are often linked to transcriptional start sites. However, since the number of CpG islands is less than the number of replication origins, GC content is unlikely the only determinant of replication origins. The methylation status of CpG islands has also been shown to affect metazoan replicator activity in both positive and negative ways [73,124]. Altogether, these studies clearly demonstrate the wide range of elements that cooperate to determine metazoan replicator activity and resulting versatility in origin usage.

Nevertheless, the main task of mammalian ORC identical to its yeast counterpart is to load replicative helicases onto DNA during the late M and G1 phases of the cell cycle [29]. The temporal gap between licensing and firing provides a time window during which dynamic events such as collision with transcription complexes can relocate the MCM double hexamer [125–128], thereby allowing for flexibility in specifying helicase loading sites. As a consequence, this may contribute to the coordination of DNA replication and transcriptional programs during development and cell fate transitions, which is crucial for the cell fate and life cycles of higher eukaryotes.

## Nucleosome positioning at replication origins

One important aspect of origin activity is the positioning of nucleosomes around replication origins. Advances in molecular biology and genomic studies have made it possible to profile the location of nucleosomes throughout the entire genome in a variety of organisms [129–132]. In budding yeast, profiling of nucleosome positioning at ARS elements in the yeast genome revealed that origins of replication are typically devoid of nucleosomes with weakly positioned nucleosomes on either side of the ARS element [129,133]. However, a close examination of the flanking nucleosomes around a more precisely identified ACS sequence within the ARS revealed well-positioned nucleosomes at almost all origins of replication [53,134]. One mechanism that likely helps to create these NFRs is the specific sequence at replication origins in yeast. Poly(dA:dT) elements, like the ones found at ARS, were shown to destabilize the interaction of histones with DNA, resulting in the preferential exclusion of nucleosomes [135–137]. More recently, it was shown that ORC plays an active role in establishing regular nucleosomal arrays at yeast replication origins [138]. By genome-wide biochemical reconstitution assays and screening of 17 purified histone chaperones and chromatin remodelers, it was shown that the Orc1 subunit of ORC in collaboration with the four chromatin remodelers INOsitol-requiring 80 (INO80), Imitation SWitch subfamily (ISW1a), ISW2 and chromatin organization modifier, helicase, and DNA-binding domains (Chd1) establishes the NFR as well as flanking nucleosomal arrays at yeast origins. Intriguingly, another study in yeast was able to ‘humanize’ the yeast ORC complex and recapitulate the nonspecific binding of ORC to NFR sites in the yeast genome [56]. In this study, a 19 aa IH located at the Orc4 subunit of the ORC complex was predicted to bind the ACS in a sequence-specific manner based on structural data. The results showed that this region indeed confers a base-specific binding property of yeast ORC and when removed leads to a much more dispersed binding pattern of ORC throughout the genome. Interestingly, newly emerged ORC binding sites are not randomly distributed but are predominantly found at NFR upstream of the transcription start sites, strongly suggesting an overall preference for ORC binding to NFRs. This is also consistent with a recent study indicating an alternative sequence-independent mechanism of ORC binding and thus origin licensing. Single molecule *in vitro* studies demonstrated that ORC not only can bind an ACS sequence but also has the cryptic capacity to bind nucleosomes near an NFR which can also ultimately lead to origin licensing in yeast [139]. These results are fully consistent with previous studies in metazoans, demonstrating that ORC binds nonspecifically to accessible, nucleosome-free DNA [82,134,140–142].

In addition, several studies indicate the importance of ORC binding in nucleosome positioning. For example, at ARS1, mutating either the ORC binding site or the Abf1 binding site results in the nucleosomes encroaching into the NDR of ARS1 from only the respective site containing the mutation. At ARS307, where no Abf1 binding site is present, mutating the ORC binding site results in the reduction of the NFR size of ARS307 from both sides [58,143]. Finally, overall nucleosome occupancy is another parameter critical to the origin function. For example, high-resolution analysis of genome-wide nucleosome positioning has identified different chromatin architectures at early and late origins, with early origins displaying a higher occupancy of nucleosomes immediately upstream (−1) and downstream (+1) from a broader NFR feature, along with better positioning of the flanking nucleosomes, compared to late origins [144]. Origins that have fewer nucleosomes tend to be more efficient and early firing, whereas a high nucleosome occupancy leads to inefficient and late firing origins [145]. In addition, the spacing of nucleosomes into regular arrays around origins positively affects replication initiation as shown in a reconstituted *in vitro* replication system [138].

Similar to yeast, mammalian replication origins are nucleosome-depleted [146]. However, analysis of nucleosome architecture around initiation sites at efficient mammalian replication origins showed high variability in nucleosome conformations. Interestingly, initiation sites at efficient CpG island-associated origins always occur at positions of high nucleosome occupancy. ORC binding sites, however, occur at adjacent but distinct positions marked by labile nucleosomes [147]. Together, it is clear that the positioning and occupancy of nucleosomes at origins affect their replication properties, but the precise chromatin states that are permissive or restrictive to efficient origin activation, how the loading of ORC and MCM2–7 complexes fit precisely into this nucleosomal landscapes and what is the heterogeneity of chromatin states at the single-molecule level are still open questions in both yeast and metazoan cells.

## Origin histone marks

**Figure 2.**
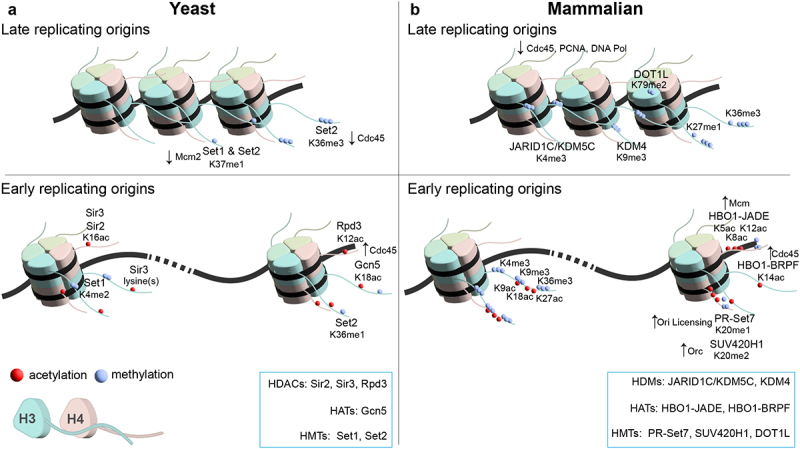
Nucleosome positioning and histone modifications at replication origins. Various histone marks associated with early and late replication origins in yeast (a) and mammalian cells (b) are shown. Histone acetylation at nucleosomes adjacent to origin generally creates open chromatin structure and is a feature of early replication origins in both yeast and metazoans. Euchromatic regions increase the probability of association with limiting replication factors like CDC45. Hypoacetylation creates compact chromatin structure and limit binding of replication factors. Various writers and erasers are illustrated on top of specific histone modifications. HDACs – histone deacetylases, HATs – histone acetylases, HMTs – histone methyltransferases, HDMs – histone demethylases.

Histones are the target substrates for a large number of posttranslational modifications (PTMs) [148], from which several have been demonstrated to influence DNA replication and origin function. As mentioned above, the origins of replication are preferentially located in an accessible, nucleosome-depleted state, allowing access of the DNA to replication factors. However, the local histone modification state of the surrounding nucleosomal arrays modulates origin licensing, RT, and efficiency in both yeast and metazoan cells (Figure 2).

### Histone modifications that regulate origin licensing and activation in yeast

In yeast, it has been shown by mass spectrometry that the acetylation states of histones H3 and H4 flanking replication origins are markedly different compared to bulk histones and also show specific patterns of acetylation. These acetylation patterns are increasing upon cell cycle release in S and G2/M phases, suggesting an important function during DNA replication. Accordingly, a quintuple mutant of these investigated histone lysine residues resulted in prolonged S phases as well as decreased replication efficiencies, suggesting that different acetylations could act together in regulating replication origin activation [149].

H4K16 deacetylation by histone deacetylase silent information regulator 2 (Sir2) leads to a reduced ability of early replication origins to load MCM proteins. This results in changes in the origin licensing landscape in such a way that origin activation is more evenly distributed throughout the S phase [150,151].

On the other hand, histone methyltransferases, Set1 and Set2, are responsible for another histone methylation mark that is only poorly characterized, H3K37me1. This modification can be found throughout the whole yeast genome. However, a significant depletion of this mark at replication origins is observed. Interestingly, H3K37me1 is able to impair the interaction of MCM2 with chromatin. Consequently, the loss of H3K37me1 leads to replication from inefficient origins and even spurious replication outside of canonical ARS sites while at the same time decreasing replication initiation from canonical replication origins [152]. At the ribosomal DNA (rDNA) locus, Sir2 was shown to repress the large majority of replication origins in the rDNA locus of yeast. As a result, only about 20% of the ribosomal ARS (rARS) are used to replicate this multi-copy locus [153], which normally consists of 150 to 200 tandem repeats with each repeat containing a rARS. It was shown that this epigenetic silencing of origin firing by Sir2 promotes genome stability by suppressing replication-dependent rDNA recombination of this repetitive locus [154].

Outside of the rDNA locus, Sir2 also controls the activation of certain origins, as a loss of Sir2 function results in persistent replication gaps during the S phase [155]. One hypothesis proposes that, by repressing rDNA replication, important initiation factors are not sequestered by the rDNA locus, so that other non-rDNA replication origins can initiate replication, which ultimately leads to a homogeneous replication of the genome [24,154,155,].

Interestingly, Sir2 alone is acting at the rDNA locus as well as some euchromatic replication origins, whereas the silent mating type loci HoMothalliam Left (HML) and HoMothalliam Right (HMR) are repressed by the combination of all four proteins of the SIR family (Sir1/Sir2/Sir3/Sir4) [156]. Telomeric regions are affected by Sir2, Sir3, and Sir4 [157]. Intriguingly, mutation of Sir3 causes telomeric origins to fire earlier, suggesting that this protein is also important for establishing the late RT at telomer-proximal origins. Consistently, transferring early replication origins to the silenced telomere region delayed their firing time significantly [158]. On the other hand, tethering Sir4 to the early firing replication origin ARS305 delayed RT at this locus [159], strongly supporting the crucial role of Sir proteins in the origin regulation at specific genomic regions.

At genomic regions that are not affected by SIR proteins, the deacetylase reduced potassium dependency 3 (Rpd3) regulates the RT of certain late origins. Rpd3-deleted cells show increased histone acetylation levels leading to earlier replication initiation at the affected origins [24,160,161]. Accordingly, targeted histone acetylation by recruiting the Spt-Ada-Gcn5-Acetyl transferase (SAGA) complex histone acetylase general control nonderepressible 5 (Gcn5) to a late firing origin also advances RT. Mechanistically, it was shown that the local increase of histone acetylation allows preferential recruitment of the limiting replication initiation factor Cdc45 [23].

In addition to acetylation events, histone methylation also affects origin function. H3K4 di-methylation, which is established by the histone methyltransferase Set1, is an important mark for proper origin function. This was shown by elevated loss rates in plasmid stability assays as well as by severe growth defects in several hypomorphic replication mutants [162]. H3K36 methylation by Set2 is another important mark that regulates the association of Cdc45 with replication origins. It has been shown that H3K36me1 can be found at early replicating origins, whereas H3K36me3 is predominantly linked to late replicating origins. This suggests that the methylation status of this residue can determine the RT by advancing or delaying the association of Cdc45 to replication origins [163]. Together, these studies illustrate that many histone PTMs play a role in origin regulation, but how they are playing together or have combinatorial roles at individual origins is still under investigation.

### Histone modifications that regulate origin licensing and activation in metazoan cells

In higher eukaryotic cells, local histone modifications also have potentially fundamental roles in shaping the replication initiation landscape. Many studies have searched for chromatin modifications preferentially associated with replication origin sequences and correlated their depletion or enrichment with replication activity [10,164–168]. These studies resulted in a general but likely simplified picture that early replicating origins are preferentially associated with histone modifications that promote open, accessible chromatin, such as H3K4me1/2/3, H3K9ac, H3K18ac, H3K36me3, and H3K27ac. Late replicating regions, however, tend to associate with H3 and H4 hypoacetylation, H3K9, and H3K27 methylationand are found in heterochromatic regions [169]. However, it is important to note that these marks neither provide an accurate prediction where replication initiates nor are they sufficient to explain the early or late timing of individual origin firing events. Emerging evidence suggests that the interplay of the replication initiation landscape and the patterns of activating or repressive chromatin marks are likely much more complex and strongly depend on genomic context, cell type, and crosstalk of the combinatorial potential of different histone modifications and their respective epigenetic modifiers.

Histone modifications affecting DNA replication can be broadly grouped into modifications that affect origin licensing in the G1 phase and modifications that affect origin activation in S phase cells. For origin licensing, the methylation state of H4K20 plays an important role. The mono-methylation is established by histone lysine N-methyltransferase PR-Set7, and the increase in H4K20me coincides with the licensing process of replication origins. During the S phase, PR-Set7 is degraded resulting in a loss of H4K20me which prevents re-licensing in the S phase. Strikingly, a non-degradable mutant of PR-Set7 resulted in re-replication and genomic instability caused by H4K20me that was maintained in the S phase [170]. Interestingly, H4K20 dimethylation was also shown to be enriched at replication origins. The ORC1 subunit of the ORC complex specifically binds H4K20me2 via its bromo-adjacent homology domain [171]. Abolishing the H4K20me2 binding properties of ORC1 resulted in impaired loading of ORC1 to replication origins as well as decreased stability, ultimately leading to defects in cell cycle progression [171]. In this regard, a recent study highlighted the role of the histone variant H2A.Z in recruiting histone lysine N-methyltransferase SUV420H1 and promoting the deposition of H4K20me2 which is then recognized by ORC1. Here, it was shown that nucleosomes containing the histone variant H2A.Z are enriched with H4K20me2 and ORC. In this proposed pathway, the histone variant H2A.Z, enriched at CpG island promoters, epigenetically regulates the licensing and thus activation of early replication origins through the SUV420H1-H4K20me2 axis [172]. Early studies of the human β-globin domain linked histone acetylation with origin activity. The human β-globin domain employs a single replication origin that fires early in S-phase in erythroid cells. However, replication is delayed to a later time point in S-phase in non-erythroid cells [173]. This was shown to correlate with the acetylation status of the origin-associated histones. Accordingly, targeting the late firing origin of the non-erythroid cells with the human acetylase binding to ORC1 (HBO1) histone acetylase could advance RT of the β-globin locus. On the contrary, deploying the histone deacetylase HDAC2 to this origin in erythroblasts led to delayed replication [174]. In this context, HBO1 is a histone acetylase that likely plays a key role in origin histone acetylation as well as proper origin licensing and activation. The HBO1 catalytic subunit can associate with different scaffold proteins, resulting in altered substrate specificity. In complex with the Gene for Apoptosis and Differentiation (JADE) proteins, this complex acetylates histone H4, whereas, with the bromodomain and Plant HomeoDomain (PHD) finger-containing (BRPF) proteins, the complex will preferentially target histone H3 for acetylation [175]. Acetylation at H4K5, H4K8, and H4K12 by the HBO1-JADE complex was shown to be crucial for replication licensing by facilitating the loading of the MCM complex [142]. In contrast, H3K14 acetylation is established when HBO1 associates with BRPF3. This histone modification is important for origin activation in the S phaseas abolishing H3K14ac by depletion of BRPF3 impairs the recruitment of CDC45 [176].

For the process of origin activation, histone methylation plays an important role in regulating mammalian origins. H3K4 methylation (H3K4me1/2/3) and H3K36me3 are linked to early, active origins, which associate with open chromatin structure, whereas H3K9 and H3K27 methylation is linked to heterochromatin and late replicating origins. Interestingly, H3K4me3, although enriched at active promoters and generally correlated with early replication, has also been suggested to repress origin firing. The histone demethylase jumonji AT-rich interactive domain 1C (JARID1C)/lysine specific demethylase 5C (KDM5C), which predominantly targets H3K4me3 for demethylation, is responsible for fine-tuning the H3K4 methylation status at origins. Loss of JARID1C/KDM5C leads to elevated H3K4me3 levels which, in turn, causes replication defects by impaired loading of CDC45 to origins [177].

Other histone modifications and their combinations may also have synergistic effects on replication initiation. For example, KDM4-mediated demethylation of H3K9me3, a mark usually correlated with heterochromatin and late replicating regions, appears to alleviate replication repression and promote chromatin association of Cdc45, proliferating cell nuclear antigen and DNA polymerases during the S phase of the cell cycle [178]. Another interesting histone modification that was shown to be enriched at replication origins is H3K79 dimethylation. Accordingly, chromatin immunoprecipitation sequencing (ChIP-seq) data revealed that 23% of the H3K79me2 peaks overlapped with replication initiation sites. Depleting Dot1-like protein (DOT1L), which is the methyltransferase responsible for H3K79 methylation, led to genomic re-replication, suggesting that this histone modification is an important mark that ensures replication only takes place once per cell cycle [179].

By studying cis-acting DNA sequences that associate with RT variation among human DNA samples, a set of histone modifications were identified at such RT quantitative trait loci that correlate with both the locations of initiation sites as well as with RT [180]. Histone methylations, in particular H3K4me3, H3K9me3, and H3K36me3, were individually repressive for replication and tended to associate with late origin activation [181]. However, the combination of all three trimethylations at the same genomic loci was strongly associated with early replication. Furthermore, the presence of these three trimethylation marks on a background of general histone tail hyperacetylation accurately predicted the locations of the majority of active replication initiation sites across three different human cell types. These seemingly contradictory results show the complex application of the histone code to origin regulation. Thus, the genomic landscape of replication initiation may be largely determined through the patterns of repressive and active chromatin marks and their combinations. It is worth mentioning that the observed histone modification landscapes could also represent by-products of the writers of the respective PTMs which might target other proteins that are associated with replication origins to enhance their function or fine-tune nucleosome positioning. This would still be consistent with all the phenotypes that can be seen after the loss of the respective enzymes, but the cause and consequence of how the presence of individual histone PTMs directly affects replication initiation are difficult to disentangle.

## Trans-acting factors and 3D nuclear organization in RT control

### Trans-acting factors in yeast and metazoan cells

In addition to DNA sequence properties, nucleosome positioning, and histone PTM landscape of replication origins, various other *trans* origin-binding factors can modulate the behavior of replication origins. Not surprisingly, the level of loaded pre-RC complex components like the MCM2–7 double hexamers itself is crucial to replication origin activity. For example, one study using ChIP-Seq of MCM subunits in yeast reported that replication origins can load different amounts of MCM2–7 helicases with early origins preferentially loading more complexes than their late counterparts [182,183]. However, other studies suggested that most origins either load exactly one or no MCM2–7 double-hexamer at all [184,185]. Thus, the precise number of MCM2–7 complexes is still under investigation. Regardless of this gap in our knowledge, it is clear that depleting cells for MCM proteins lead to drastic changes in the RT of distinct replication origins [183], suggesting that balanced cellular levels of MCM proteins is an important criteria for replication regulation. Interestingly, multiple origin-binding factors including the ORC complex were shown to be sumoylated in both yeast and humans [186,187]. A recent study demonstrated that an ORC hypersumoylation mutant preferentially reduced the function of a subset of early origins by reducing MCM chromatin association *in vivo*, suggesting that this protein modification can fine-tune origin licensing of individual origins [188]. Interestingly, not only components of the origin licensing machinery but also factors that are required for the MCM2–7 helicase activation in the firing process are able to influence the RT program. In yeast, six factors of this process are in low abundance in the cell, namely the regulatory subunit of DDK Dbf4, as well as the activation factors synthetically lethal with Dbp11–1 2 (Sld2), Sld3, Sld7, Dbp11, and Cdc45 [26,27]. The association of these low-abundance factors is crucial for determining the order of replication initiation events, since origins must compete for these limiting factors and, generally, early replication origins will associate with these factors before late replication origins, setting them up for early activation. Consistent with this notion, a recent study used a conditional system in budding yeast to simultaneously overexpress the six firing factors and achieved a global early replication of the majority of origins in a single cell cycle [189]. The major substrate of DDK is the MCM2–7 double hexamers, and its phosphorylation by DDK is crucial for origin activation. In this context, two trans-acting factors, forkhead transcription factor 1/2 (Fkh1/2) and Rap1 interacting factor 1 (Rif1), have been well studied to work in opposing pathways to regulate early and late global RT, respectively. In brief, Rif1 helps to set up late RT by recruiting the phosphatase PP1 to origins and reverse MCM phosphorylation, whereas Fkh1/2 establishes early RT by recruiting the licensing factor Dbf4 to early origins.

In more detail, Fkh1 and Fkh2 were first identified as global determinants of replication origin timing by stimulating the early RT of ~100 replication origins. These forkhead-regulated origins are typically enriched for Fkh binding sites adjacent to the ACS and, consequently, disrupting this motive also leads to a deceleration in the respective RT. A similar result was obtained when examining the RT in fkh1/2 knock-out mutants, where the early firing of these forkhead-regulated origins was delayed [190,191]. Additionally, overexpression of Fkh1/2 resulted in a global advancement of RT, which demonstrates the stimulatory function in origin activation [192]. On a molecular level, Fkh1/2 recruits during G1 phase one of the limiting factors, Dbf4, to the respective origins by direct interaction. This, in turn, leads to the association of Cdc45, which facilitates relocation from the nuclear periphery and clustering of early origins in the nuclear interior [193,194]. Intriguingly, the activity of Fkh1 is also linked to the previously mentioned histone deacetylase Rpd3, as a recent study revealed that Rpd3 activity hinders the binding of Fkh1 and thus counteracting Dbf4 recruitment for early activation of the respective origins [195].

Besides these forkhead-activated replication origins, origins near centromeres also typically show an early RT [8]. In a similar mechanism, the chromosome transmission fidelity 19 (Ctf19) complex, which is a component of the kinetochore, is able to recruit Dbf4 from telophase until G1 phase. Subsequently, the Sld3 and Sld7 initiator proteins are recruited to these replication origins which allow for the association of Cdc45 and, ultimately, early replication in the subsequent S phase [196].

In contrast, replication origins at ‘heterochromatic’ telomeric and subtelomeric regions often replicate late or are passively replicated in the S phase. Deletion of the telomere-associated Rif1 protein leads to an abnormal early replication of telomeric and telomere-proximal regions [197,198]. Mechanistically, it was shown that Rif1 interacts with the phosphatase PP1/Glc7, which is able to counteract premature phosphorylation events by DDK in the G1 phase [199–201]. The specificity toward telomeric regions is achieved by a second interaction of Rif1 with the repressor-activator protein 1 (Rap1), which is a preferential telomere-binding protein. Briefly, Rap1 sequesters the limited amounts of Rif1 to telomeric regions and thus creates high concentrations of this protein at the chromosomal ends. Interestingly, disturbing the Rif1 telomere interaction by mutation of its Rap1-binding domain leads to the recruitment of Rif1 to non-telomeric origins [202]. Besides the telomeric origins, a large fraction of the replication origins at the rDNA repeats is also regulated by Rif1. This helps to limit the number of active replisomes which, in turn, increases genome stability at this repetitive locus [203]. Detailed biochemical analysis shows that Rif1 is a G4 binding protein, and its binding to multiple G4 assemblies through its C-terminal domain helps form chromatin loop structures and regulate origin timing in these looped regions [204–206].

Rif1 has also been identified as a global regulator of RT in both human and mouse cells [207,208]. Depleting Rif1 in HeLa cells led to early activation of mid-S phase origins and, in turn, to a significant delay of some early S phase origins [209]. Mechanistically, human Rif1 also interacts with the PP1 phosphatase to counteract DDK activity similar to yeast. In addition, mouse Rif1 is an essential factor as Rif1 deficiency leads to both defective G1/S transition and chromatin re-organization after DNA replication. As a consequence, the RT program is strongly perturbed as both early- and late-replicating domains are instead replicated in the mid-S phase or randomly throughout the S phase [207]. However, contrary to its inhibitory role in origin activity, Rif1-PP1 is also important for origin licensing in humans. By preventing phosphorylation of Orc1 in the G1 phase, it was shown to block degradation and stabilize Orc1 in the G1 phase, thereby stimulating origin licensing [210].

Another factor that influences origin activity is the replication initiation determinant protein (RepID). RepID binds a subset of replication origins in mammalian cells and is required for replication initiation at these origins. Accordingly, the depletion of RepID leads to decreased initiation frequencies as well as slower fork elongation and stalling events [211]. Further studies showed that RepID is necessary for recruiting the cullin 4 RING E3 ubiquitin ligase (Crl4) to this set of replication origins. This complex leads to ubiquitination and subsequent degradation of different replication-associated factors such as the Cdt1 chaperone, possibly disturbing subsequent initiation steps. Interestingly, RepID associates and exerts its function mainly at early replication origins. On a subset of late replication origins, however, the Skp1, Cullins, F-box (SCF) ubiquitin ligase complex, which degrades the same proteins, is recruited by S-phase kinase-associated protein 2 [212].

Together, this large amount of origin-interacting factors could support the idea that distinct sets of replication origins interact with certain regulatory proteins to modulate their activity in a subset-specific manner. This could also help to fine-tune replication in different cell types or stages in development in order to match replication with the changes in the transcriptional landscape of the corresponding cell type.

## Spatial organization of replication origins in yeast

**Figure 3.**
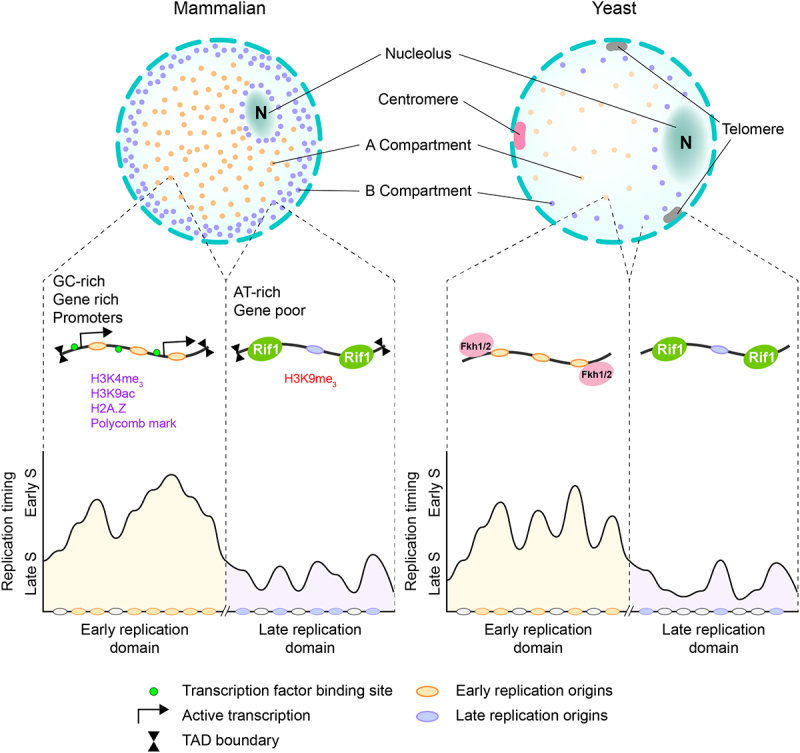
Nuclear organization of replication origins. Genome organization of replication domains in (a) mammalian and (b) yeast. In both models, early replicating domains are clustered in the center region while late replicating domains are at peripheral region. (a) In metazoans, early replication domains are gene-rich, GC-rich, and enriched with regulatory elements like promoters and enhancers, the TAD boundary is insulated by CTCF. Late replicating domains are AT-rich and gene-poor. (b) In yeast, early replicating domains are mediated mainly by Fkh1/2, the late replicating domains are located at telomeric regions and mediated by Rif1.

Another important aspect that impacts the RT program is the spatial organization of replication origins within the nucleus (Figure 3). However, a distinction has to be made between the overall folding of chromosomes in the nucleus and the relative position of replication origins in reference to distinct nuclear substructures. In yeast, both centromeres and telomeres can be found at distinct and reproducible positions inside the nucleus [213]. While centromeres are positioned in proximity to the spindle pole body, telomeres are positioned at the nuclear periphery, away from the centromeres. While telomeres generally display late RT, it has been shown that this is not caused by the peripheral location itself. For example, after disrupting the positioning of telomeres from the nuclear periphery, replication origins still kept their late RT [214]. Similarly, tethering an early replication origin to the nuclear periphery did not change its early replication profile [215], arguing that, in these cases, other mechanisms are dominant and that subnuclear localization alone is not sufficient to determine replication initiation.

However, the overall genomic organization of how chromosomes are folded within the nucleus has a stronger impact on the replication profile as compared to the relative position within the nucleus. The existence of replication foci was demonstrated in budding yeast both *in vitro* [216] and *in vivo* [217]. Notably, this live cell imaging approach also provided strong evidence that, after origin firing, the two sister replisomes stay associated within these foci and that the double-stranded DNA products are extruded as loops from two replisomes. Furthermore, super-resolution microscopy discovered that budding yeast replication factories typically consist of one up to four sister replisomes. Interestingly, the decision of which replication start sites cluster together is mainly a stochastic process, meaning that the number and type of replisomes which cluster together can vary from cell to cell [5]. However, a preference is observed that early origins that are in the close neighborhood on the linear chromosomes show a higher frequency to cluster together [217,218].

Several molecular mechanisms have been described in how such clustering of replication origins can be achieved in the yeast nucleus. At centromeric regions, replisomes of different chromosomes are brought in close proximity by the tethering of the kinetochores to the spindle pole body, which might help to organize these replisomes into a centromeric replication factory [196]. Second, the previously mentioned Fkh1/2 transcription factors were shown to cluster individual replisomes together in order to form replication factories [190]. This is due to the ability of Fkh1/2 to form homodimers, with each protein being able to bind a different origin and ultimately bridging them in close proximity [219]. Another important aspect of this interaction is a relocation event within the nucleus. Precisely, upon Fkh1 and Dbf4 activation in the G1 phase, Fkh-activated origins relocalize from the nuclear periphery to the nuclear interior [194]. A recent study developed a single-locus chromatin proteomics approach to specifically excise and purify replication origins and determined the chromatin composition of distinct early-efficient and late-inefficient replication origins. Interestingly, the microtubule-encircling associated with spindles and kinetochores (Ask1)/DASH complex co-purified with specific replication origins. Using genetic perturbations, Ask1/DASH was shown to regulate the timing of more than 100 replication origins in the yeast genome. Thus, the connection between RT, chromosomal organization, and the microtubule cytoskeleton could provide another structural framework for sub-nuclear rearrangement of origins into early-efficiently firing replication factories and explain how this movement of individual genomic loci is physically achieved through the selected attachment of microtubules at specific origins [68].

One potential benefit of origin clustering might be to increase the local concentration of replisome components. For example, the efficiency of replication initiation can be increased, since the limited pool of initiation factors can be concentrated on these discrete sites and, therefore, facilitate the early firing of the clustered origins. Furthermore, this might also be particularly helpful for coping with replication stress or fork stalling within this replication factory, since the initiation factors could be efficiently recycled to a neighboring replication start site and otherwise dormant origins could be activated in order to finish replication of the whole genomic region within the replication factory [220].

## Spatial organization of replication origins in metazoan cells

Early studies in mammalian cells have shown that, during S phase, replication takes place at discrete replication foci within the nucleus [221]. Such replication foci have later been referred to as replication factories, since several replisomes act together within these foci to duplicate the genome in a synchronous manner [222,223].

In human cells, it was first speculated that replication foci consist of 10–100 replisomes [224]. Such replication domains have been further resolved by superresolution microscopy, showing that they are typically 150 nm in size and contain one spatially-separated single replisome up to four co-replicating regions [225]. This also challenged the original model of replication factories as distinct entities where bulk replication of several replisomes takes place [226]. Zooming out from individual replisomes, replication domains were defined as clusters of replication foci firing at the same time in the S phase. Mammalian replication domains were first described using microarray-based approaches with very low resolution [227]. These approaches were extended genome-wide by tiling arrays [228] and next-generation sequencing [180,229,230]. Large chromosomal domains were further identified and found to replicate at specific times during S-phase [166,231]. From these studies, it is clear that the mammalian genome is compartmentalized into broad regions with distinct RT in early, mid, or late S-phases [232,233].

In addition, genome-wide chromatin interaction maps generated via Hi-C [234] revealed strong correlations between the DNA RT program and nuclear architecture [235]. Importantly, the boundaries of topologically associated domains (TADs) [236] frequently separate RT domain boundaries [237]. The mechanisms responsible for establishing and maintaining TADs are actively investigated. Particularly, the insulator element CCCTC-binding factor (CTCF) is enriched at the borders of TADs [236], and ablation of a specific CTCF site, for example at the Hox gene cluster, results in an expansion of permissive chromatin and disruption of a TAD boundary [238]. This disruption causes the Hox genes to enter an active domain and become subject to transcriptional activation. Early-replicating domains are typically found in such transcriptionally active regions in the center of the nucleus. They are frequently associated with activating chromatin modifications and histone variants, including H3K4me3, H2A.Z, and H3K9ac, and are enriched for GC content, genes, and regulatory elements (promoter and enhancer sites). Regulatory elements contribute to an accessible chromatin environment permissive to ORC binding [79,85]. In addition, the majority of protein-coding genes are located in early-replicating domains, suggesting that there is evolutionary pressure to ensure that protein-coding genes are duplicated early in the S phase to avoid the increased accumulation of mutations in late-replicating domains [239,240].

In contrast, late-replicating domains are associated with the nuclear periphery and colocalized with lamin-associated domains (LADs) [241]. Association with the nuclear periphery may reduce the accessibility of critical kinases (DDK and CDK) and/or limiting initiation factors such as CDC45. Notably, TAD structures more frequently exhibit an early RT, whereas LADs are typically late replicating [242]. However, deletions of the TAD boundaries had little to no effect on the RT of these domains. Instead, cis-regulatory elements have been found to heavily modulate RT within these regions alongside with A/B compartmentalization, TAD structure, and transcription [243]. Taken together, these findings suggest that genomic architecture also plays important role in RT. But still, it is not known whether this organization into specific domains only serves to coordinate the simultaneous replication of different genomic parts and that RT is a consequence of other factors discussed above, or if there is also a functional aspect through this organization that dictates the replication within these domains.

## Conclusions/outlook

Over the last decades, DNA replication has been an intensive field of research. Much effort has been made to unveil the principles of how replication is initiated at these particular genomic loci as well as the determinants of origin firing. The earliest biochemical reconstitution of the origin licensing system in *Xenopus laevis* demonstrated that ORC, CDC6, CDT1, and MCM2–7 can promote functional origin licensing and the assembly of MCM2–7 complexes onto *Xenopus* sperm nuclei [244]. Apart from these early studies in animal cells, the yeast *S. cerevisiae* turned out to be a powerful model organism to study the precise mechanism of replication initiation. In particular, the successful biochemical reconstitution of origin-dependent eukaryotic DNA replication *in vitro* using purified budding yeast proteins demonstrates that all key components of this process have been identified [245]. This *in vitro* system has been further refined and yielded detailed insights into how replication is affected by chromatin [246,247] and by non-essential replisome components [248]. However, many different factors have been found to influence origin firing ranging from DNA sequence features, nucleosome positioning, histone modifications, or other *trans-acting* chromatin factors, which remain difficult to reconstitute *in vitro*. Therefore, the detailed molecular mechanisms of how these factors regulate the replication program *in vivo* remain elusive. Most likely, a complex hierarchical interplay of all these described factors is crucial, which can lead to the association of limiting replication factors to respective origins. Also, there is a high possibility that more unknown factors are able to influence the replication program. An *in vitro* replication system of native replication origin domains purified from yeast [68] would be a perfect model to directly test the role of chromatin in regulating replication efficiency. In contrast, replication origins of higher eukaryotes have been more difficult to analyze since the exact position in the genome is not defined. However, advancements in long-read sequencing and imaging methods such as the newly established ORM method show great promise to providing higher resolution mapping of origins in metazoans.

So far, robust characterization of replication origins is mainly performed in a population-based manner. However, it is well documented that the replication of eukaryotic genomes is highly stochastic [3–5]. Population-based studies will average the heterogeneous replication initiation events within a cell population [7,8]. Recent single-molecule based sequencing approaches in budding yeast generated whole genome maps of replication dynamics and discovered a class of low-frequency stochastic origins in budding yeast [101]. In the future, mapping replication origins using single-molecule approaches will reveal more insights into cell-to-cell origin heterogeneity. With such new methods in hand, characterizing changes in replication origin usage in different cell types at different developmental stages will provide new insights into the plasticity of the replication program during programmed development and disease transformation.

Box 1. Methods for mapping/identification of replication origins.

**Figure:**
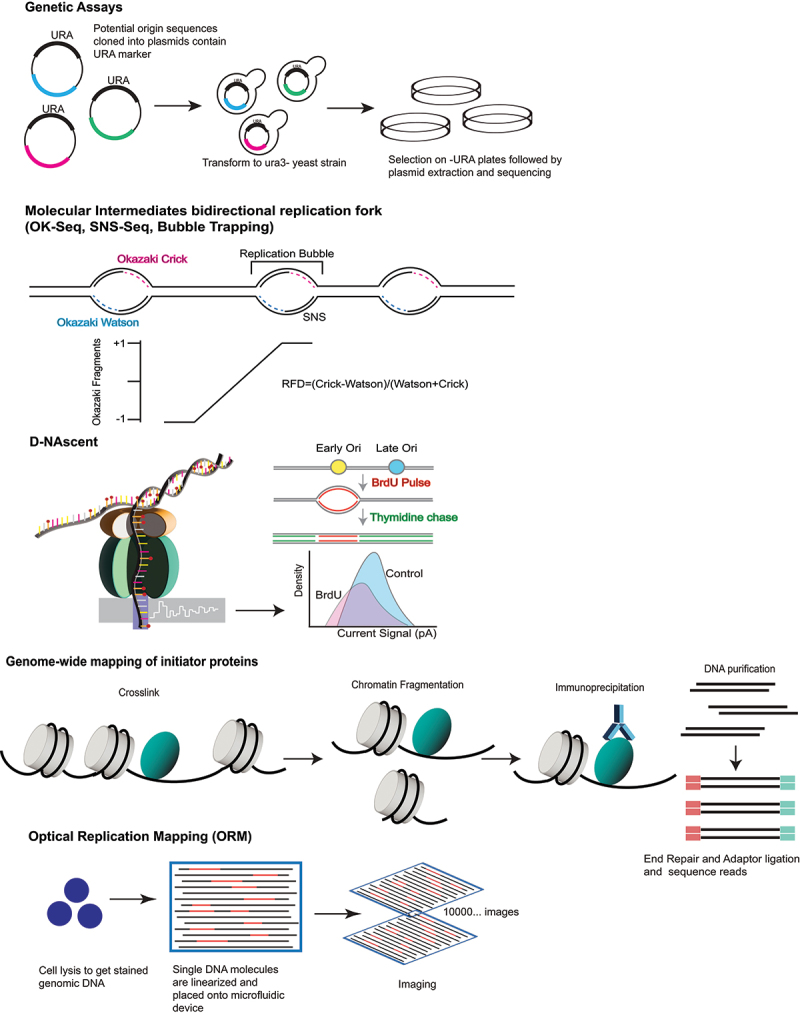

Early work in mammalian cells used tritium-labeled thymidine and fiber autoradiography to directly visualize the size distribution of replisomes resulting from bidirectional DNA replication [91]. Throughout the years, the advance in molecular biology and next-generation sequencing allows more robust identification of start sites of DNA replication in eukaryotes. Major classic and up-to-date methodologies for origin identification are listed.

**Genetic assays**

In budding yeast *S. cerevisiae*, cis-acting replicator elements were first identified by their ability to ensure the long-term propagation and maintenance of episomal DNA. Functional screens identified hundreds of short DNA sequences, termed the ARS elements, that were necessary for the duplication and maintenance of the plasmid in every cell cycle [36].

**Genome-wide mapping of initiator proteins**

Chromatin immunoprecipitation (ChIP) coupled with microarrays or next-generation sequencing has been a powerful approach for mapping the binding sites of transcription factors and DNA-binding proteins throughout the eukaryotic genomes [92]. Genome-wide mapping of ORC and MCM protein complex binding sites revealed potential origin locations. Budding yeast ORC was one of the first initiator complexes to be mapped [93]. Despite the early successes in mapping ORC-binding sites in *S. cerevisiae* and *Drosophila* [83,93], it has been a challenge to identify ORC-binding sites in mammalian systems. Despite the efforts of many laboratories, comprehensive genome-wide ORC mapping has become available only recently [79,85]. In addition, ORC has other functions independent of DNA replication such as its localization to heterochromatin [94,95,]. To achieve higher specificity, ORC ChIP data can be filtered for peaks that coincide with MCM ChIP peak [96], but this mapping of initiator proteins only reveals information about licensed origins but not reveals the subset chosen later for origin firing.

**Molecular intermediates of bidirectional DNA replication (Okazaki fragments, nascent strands, bubbles)**

A comprehensive mapping of the strand-specific distribution of Okazaki fragments throughout the genome provides the precise location of replication initiation sites as well as the direction of replication fork movement. In a landmark study, Smith and Whitehouse [97] provided the first genome-wide map of Okazaki fragments in budding yeast. Okazaki fragments were enriched by the inactivation of DNA ligase I and subsequently purified from asynchronous cells. The strand-specific nature of the Okazaki fragments was preserved during high-throughput sequencing. DNA replication origins were identified by the sharp transition in stranded-ness (Watson to Crick) surrounding an origin.

Adaptation of SNS capture assays to genome-wide approaches offers a complementary approach to mapping Okazaki fragments [98,99]. Size-selected nascent strands were enriched and purified by treatment with excess λ-exonuclease before hybridization to microarrays. The latest SNS-Seq methods yield high-resolution maps of metazoan replication origins [88,89,90,].

The initiation of DNA replication results in a 2N ‘bubble’ replication intermediate. The reduced electrophoretic mobility of these bubble structures provided a mechanism to enrich replication initiation events across the genome by trapping them in agarose plugs during gel electrophoresis [100].

**D-Nascent**

The recently developed D-Nascent nanopore sequencing method [101] allows the measurement of replication fork dynamics on both single molecules and genomic scales by detecting nucleotide analog signal currents on long nanopore traces. D-Nascent can detect 5-bromodeoxyuridine (BrdU) incorporation in the *Saccharomyces cerevisiae* genome to reveal the DNA sequences replicated during a pulse-labeling period. Under limiting BrdU concentration, it can detect the differences of BrdU incorporation to detect the active replication initiation sites, fork direction, and pausing regions.

**Optical replication mapping (ORM)**

ORM is a recently developed single-molecule technique to investigate the spatial and temporal distribution of origin firing that combines the Bionano Genomics approach to mapping long individual DNA molecules [102] with *in vivo* fluorescent nucleotide pulse labeling [103,104] to directly visualize sites of replication initiation within human cells. This approach provides good signal-to-noise characteristics and deep, genome-wide coverage, allowing the identification of rare initiation events in as few as 0.1% of human cells [105].
